# Reproducibility and diagnostic accuracy of pocket-sized ultrasound devices in ruling out compensated cirrhosis of mixed etiology

**DOI:** 10.1007/s00330-022-08572-2

**Published:** 2022-03-03

**Authors:** Andrea Costantino, Alessandra Piagnani, Nicoletta Nandi, Valentina Sciola, Marco Maggioni, Francesca Donato, Maurizio Vecchi, Pietro Lampertico, Giovanni Casazza, Mirella Fraquelli

**Affiliations:** 1 Gastroenterology and Endoscopy Division, Foundation IRCCS Ca’ Granda Ospedale Maggiore Policlinico, Milan, Italy; 2 University of Milan, Milan, Italy; 3 Pathology Division, Foundation IRCCS Ca’ Granda Ospedale Maggiore Policlinico, Milan, Italy; 4 Gastroenterology and Hepatology Division, Foundation IRCCS Ca’ Granda Ospedale Maggiore Policlinico, Milan, Italy; 5 CRC “A. M. and A. Migliavacca” Center for Liver Disease, Department of Pathophysiology and Transplantation, University of Milan, Milan, Italy; 6 Department of Biomedical and Clinical Sciences, “L. Sacco” Hospital, University of Milan, Milan, Italy

**Keywords:** Liver diseases, Liver cirrhosis, Point-of-care systems, Ultrasonography

## Abstract

**Objective:**

Fibrosis is the key prognostic factor in chronic liver disease patients. Liver surface nodularity (LSN) is the ultrasonographic sign with the highest accuracy to detect advanced liver fibrosis. The use of pocket-sized ultrasound devices (PUDs) has been assessed in several clinical settings but never as regards chronic liver disease (CLD) severity. Our study aimed at evaluating the feasibility, reproducibility, and diagnostic accuracy of PUD in LSN identification.

**Methods:**

We enrolled all the consecutive adults referred for percutaneous liver biopsy. Two independent operators evaluated LSN by PUD; one sonographer used standard ultrasound (US). Transient elastography (TE) and liver biopsy were performed on all the patients. PUD reproducibility was evaluated by Cohen’s *k* statistic. PUD, standard US, and TE results were compared with histology staging.

**Results:**

A total of 104 consecutive patients (aged 54** ± **14 years) with mixed-etiology CLD were studied. Assessment by PUD was feasible in all the patients and showed very good inter-observer agreement with Cohen’s *k* = 0.87 (95% CI 0.72–0.95). The diagnostic accuracy estimates for PUD in diagnosing compensated cirrhosis (*F* = 4) were 87.5% sensitivity, 76.8% specificity, positive likelihood ratio (LR) 3.78, and negative likelihood ratio (LR-) 0.16, while those for standard US and TE (> 12.5 kPa) were, respectively, 87.5% sensitivity, 72.6% specificity, LR+ 3.2, and LR- 0.17, and 87.5% sensitivity, 90.5% specificity, LR + 9.2, and LR- 0.13.

**Conclusions:**

PUD reproducibility in assessing LSN was excellent even with operators of different experience. PUD performed very well in excluding advanced CLD. PUD can be used as a first-line tool for screening patients to undergo more invasive techniques, thus shortening the time for clinical decision-making.

**Key Points:**

• *PUD is highly reproducible in assessing the sign of liver surface nodularity.*

• *PUD showed high diagnostic accuracy in excluding the presence of advanced chronic liver disease.*

• *PUD can be used as a first-line tool for screening patients with CLD who should undergo more invasive techniques.*

**Supplementary Information:**

The online version contains supplementary material available at 10.1007/s00330-022-08572-2.

## Introduction

The need for rapid answers to clinical problems has led to the development of point-of-care pocket-sized ultrasound devices. The basic ultrasound approach with these pocket-sized devices has been defined by the European Federation of Ultrasound Societies in Medicine and Biology (EFSUMB) as “EchoScopy” to distinguish them from standard ultrasound. The EchoScope provides conventional B-mode and color Doppler imaging (CDI) and can be used for bedside assessment of patients in a variety of clinical settings, allowing clinicians to have immediate visual correlation with physical exam results.

Many small and lightweight devices with high-quality imaging are nowadays available, and the inevitable loss of resolution using these tools is offset by the possibility of obtaining immediate support to diagnostic hypotheses and therapeutic decisions [1].

To address the growing interest in this field, many recently published studies describe the usefulness of a pocket-sized ultrasound device (PUD) examination in emergency, clinical, or general practice settings, improving the allocation of more complex and expensive resources and answering common clinical questions [2–7].

In Gastroenterology and Hepatology, PUD is currently part of clinical practice to help solve questions and assist invasive procedures: PUD has proved useful for the initial screening of outpatient patients complaining of intestinal symptoms [2].

In the chronic liver disease (CLD) context, preliminary reports indicate that PUD is a reliable tool for ascites assessment, so it is potentially a reliable first-line screening technique in this context for shortening clinical evaluation [2, 8].

Despite the growing interest and technological innovation in this context, no study has been published yet to evaluate the usefulness and reliability of these devices in the assessment of liver surface nodularity.

Liver biopsy is the reference standard to evaluate liver fibrosis, but since it is an invasive procedure, there is an increasing need for non-invasive diagnostic tools capable of assessing the progression of fibrosis in CLD. These instruments need to have good diagnostic accuracy.

There are two main groups of non-invasive methods to assess fibrosis: a first group consists in the use of serological markers that can be measured in the blood, a second group uses imaging techniques, such as ultrasound, MRI, and CT, and such methods as TE [9–21]. The advantages of these tests are that they are not invasive and there are limited sampling errors and little subjectivity in result evaluation.

LSN has shown different diagnostic accuracies in predicting severe hepatic fibrosis or compensated cirrhosis. In general, it is deducted that LSN sensitivity is between 54 and 92% and specificity between 80 and 95% in the identification of compensated cirrhosis [14].

The aim of this prospective study was to evaluate the reproducibility of PUD as a tool for assessing the sign of liver surface nodularity. The results obtained were compared to those from standard ultrasound technique and transient elastography using liver biopsy as the reference standard.

## Material and methods

For inclusion in this study, we evaluated the patients with CLD consecutively admitted to our Gastroenterology and Hepatology Division between October 2018 and January 2020 to have liver biopsy performed for diagnostic or prognostic/therapeutic purposes.

We excluded patients with decompensated chronic liver disease, patients with previous liver transplantation, patients who came to perform a biopsy to a focal liver lesion, < 18-year-old patients, patients who did not give their informed consent, and patients with contraindications for percutaneous liver needle biopsy.

All the eligible subjects were enrolled after obtaining their signed informed consent and after discussing the purpose and procedures of the study.

Socio-demographic and clinical data were acquired for all the patients, including demographic data, weight, height and body mass index (BMI), indication for liver biopsy (diagnostic or prognostic/therapeutic in the context of an already known liver disease), signs and symptoms, clinical history, co-morbidity, medications taken, and any allergies. Alcohol intake was also reported to define the risk factor of CLD.

The sign of LSN was assessed by a high-frequency probe using both VScan DualProbe® (General Electric Healthcare) and by traditional ultrasound equipment, Philips iU22 x Matrix® (Philips); subsequently, the assessment of liver stiffness was performed by transient elastography with Fibroscan® (Echosens). Ultrasound (PUD and traditional US) and TE operators were blinded to each other’s results and to clinical and bio-chemical findings.

### PUD and traditional US examination

Before liver biopsy performance, every patient underwent both PUD and standard US examination to search specifically for liver surface nodularity (LSN), which was assessed by means of a 5–12-MHz transducer to examine the whole liver surface and the 2–3-cm outer layer of the liver parenchyma. A nodular aspect of the liver surface results from the effects of fibrosis and the regenerative nodules on the liver capsule. The sign was searched for in both the right (intercostal scan) and the left (subcostal scan) lobes and, according to a previous study by our group [14], it was considered to be positive if, instead of a straight and regular hyperechoic line, the liver surface appeared as a dotted or irregular line and/or the liver parenchyma was not homogeneous but showed areas of different echogenicity, reflecting underlying nodularity.

Assessment by PUD was firstly performed by an inexperienced operator (a first-year resident GE with a 10-day PUD training), blinded to the patient’s clinical and laboratory tests.

Then PUD examination was carried out by a second expert operator (with > 1000 US performed) who was blinded both to the result of the patient’s clinical and laboratory data and to the PUD results obtained by the first operator.

At the end of the examination, the second operator filled the LSN evaluation form in, and then performed the examination by standard ultrasound equipment for LSN evaluation.

**Fig. 1:**
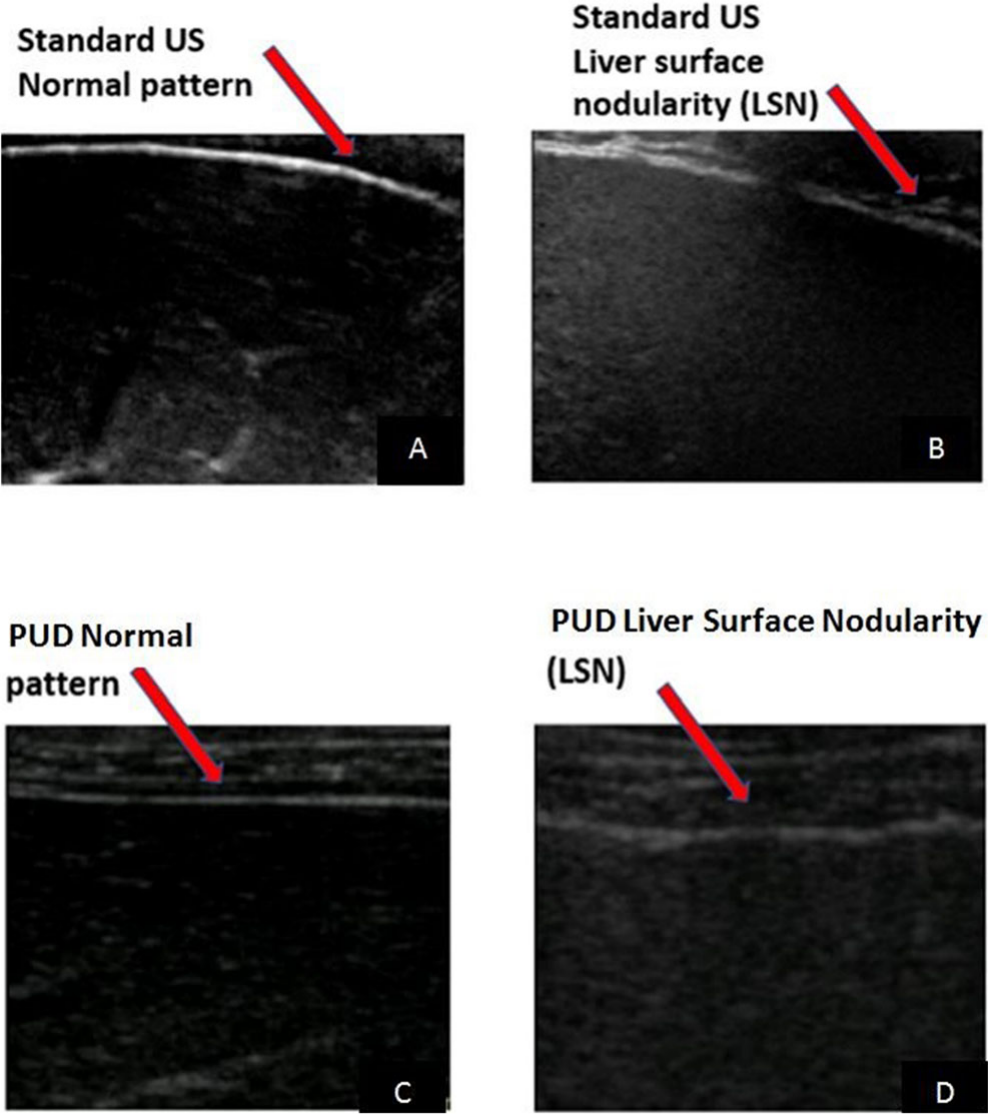
Standard US and PUD scans obtained with a 5–12-MHz transducer. **A** Normal pattern: linear liver surface with normal homogeneous parenchyma. **B** Liver surface nodularity: liver surface appears as a dotted or irregular line and liver parenchyma shows areas of different echogenicity reflecting the underlying nodularity. **C** Normal pattern: linear liver surface with normal homogeneous parenchyma. **D** Liver surface nodularity: liver surface appears as a dotted or irregular line and liver parenchyma shows areas of different echogenicity reflecting the underlying nodularity

Figure 1 shows the normal linear liver surface pattern visualized respectively by standard US and PUD.

### Transient elastography

The right liver lobe was accessed through an intercostal space while the patient was lying in the dorsal decubitus position with the right arm at maximum abduction. By FibroScan ultrasonography (US) guide, a portion of liver of at least 60-mm thickness, free of large vessels, was identified for examination. The results were expressed as the median value of the total number of measurements. The success rate was calculated as the number of validated measurements divided by the total number of measurements.

According to the EASL guidelines [15], we have made use of the XL probe instead of M probe in those patients whose BMI was > 30 kg/m^2^ or if the skin-to-liver-capsule distance was > 25 mm. In addition, the latest versions of the FibroScan machine now include the APS tool, which indicates which probe should be used for LSM. In practice, the APS tool indicates “M” or “XL” on the FibroScan screen when the probe is placed on the skin.

The results were expressed in kiloPascal (kPa). The median value was considered representative of the elastic modulus of the liver. Repeated measurements were performed until at least 10 valid values were obtained, for which the software could calculate the median value with a success rate of at least 60% and an interquartile range (IQR), which had to be less than one-third the median value of the measurements in order to be considered acceptable.

According to the results from a previous study by our group [16], the following cutoff values were used to determine liver fibrosis stage: > 7.9 kPa for the diagnosis of significant fibrosis (*F* ≥ 2), > 10.3 kPa for severe fibrosis (*F* ≥ 3), and > 12.5 kPa for cirrhosis.

### Histology

Liver biopsy was performed by an experienced hepatologist with a 18-gauge Menghini needle (Biomol; Hospital Service) under US guidance. The liver tissue was fixed in formalin and embedded in paraffin. Slices of liver tissue, 5-mm thick, were stained with H&E and Masson trichrome, and were examined by an expert liver pathologist (M.M.), who was blinded to the results of PUD, standard US, TE, and clinical data. Only those samples with 15-mm length and including at least 12 complete portal tracts were considered adequate. Liver fibrosis was semi-quantitatively evaluated and staged on a 5-point scale according to METAVIR (F0, no fibrosis; F1, portal fibrosis without septa; F2, portal fibrosis and few septa; F3, numerous septa without cirrhosis; F4, cirrhosis).

Necro-inflammatory activity (A) was also graded on a 4-point scale (A0, none; A1, mild; A2, moderate; A3, severe). The length of each liver specimen (in millimeters) and the number of fragments were recorded. Steatosis in the liver specimen was arbitrarily graded from 0 to 3 according to the percentage of fatty hepatocytes (0, < 5%; 1, 5–24%; 2, 25–49%; 3, > 50%). The length of each liver specimen (in millimeters) and the number of fragments were recorded.

### Statistical analysis

Cohen’s kappa statistic was used to assess the agreement between observers as regards the evaluation of qualitative variables, surface nodularity, and homogeneity of the parenchyma, assessed by PUD. The level of concordance between PUD and traditional US results in the identification of these two signs was also calculated. By comparing the results, slight agreement was defined for *k* = 0.00–0.20; discrete for *k* = 0.21–0.40; moderate for *k* = 0.41–0.60; substantial for *k* = 0.61–0.75; and practically full for *k* = 0.76–1 [17].

As regards the surface nodularity sign, assessed by PUD and standard US, sensitivity, specificity, positive and negative likelihood ratios (LR + and LR- with their respective 95% confidence intervals (95% CI)) for the diagnosis of advanced fibrosis (F3–F4).

The diagnostic accuracy estimates for each index test (PUD, standard US, and TE) in excluding or confirming advanced fibrosis (F3–F4) were compared and also compared to the results from liver biopsy, taken as the reference standard for fibrosis staging.

Any technically inadequate results of the techniques examined were treated on the basis of an intention-to-diagnose principle (diagnostic false negative or false positive in the presence or absence of the disease).

The study was written according to the STARD 2015 guidelines (Supplementary file).

## Results

In total, 118 patients were consecutively considered for inclusion in the study, of whom 14 were excluded because they had previously undergone liver transplantation. The remaining 104 patients met the selection criteria for our study and were enrolled: 56 women (54%, mean age: 51 ± 14 years, range 18–75 years). Their demographic, laboratory, and etiological data are summarized in Table 1.

**Table 1:** Main demographic and clinical characteristics of 104 consecutive patients with chronic liver disease who underwent PUD, standard US, TE, and liver biopsy to diagnose severe fibrosis/cirrhosis

**Variables**	**Median (range)**
Age (years)	54 (18–75)
**Laboratory**	
AST (U/L)	55.5 (16–1235)
ALT (U/L)	64 (11–1471)
ALP (U/L)	110 (34–844)
Platelets (/mm^3^)	217,500 (86,000–441,000)
INR Direct bilirubin	1.03 (0.8–1.8) 0.6 (0.17–25.17)
**Etiology**	***n*** **(%)**
NAFLD/NASH	29 (27.9%)
HBV	10 (9.6%)
HCV	2 (1.9%)
AIH	17 (16.3%)
Vascular/veno-occlusive disease	14 (13.5%)
PSC/PBC/AIH/overlap	18 (2.9%)
Alcoholic	1 (1%)
Others	13 (12.5%)

LSN assessment by PUD proved feasible in all the patients for both operators as was standard US. The inter-observer agreement on PUD assessing the presence of LSN was almost perfect with a Cohen’s *k* value of 0.87 (95% CI 0.72–0.95).

TE was successful in all the patients and no failures or indeterminate results (SR < 10 valid measurements, IQR > 30% of the median value) were observed. The mean hepatic stiffness value was 9.1 ± 6.98 kPa, with a median of 7.5 kPa (range 3.3–49.8 kPa).

Liver biopsy was successfully performed on all the patients, yielding liver specimens of 24.7 mm (range 15–48), with 85% of the specimens being longer than 20 mm. For the final evaluation, one liver sample (1%) was inadequate. At histology, 22 patients (21%) showed no signs of fibrosis (*F* = 0), 29 patients (28%) had mild fibrosis (*F* = 1), 24 patients (23%) showed significant fibrosis (*F* = 2), 20 patients (19%) had advanced fibrosis (*F* = 3), and the remaining 8 (8%) had signs of cirrhosis (*F* = 4). For the purposes of our study, we identified two groups of patients, 28 with severe fibrosis (*F* ≥3) and 75 with absence of severe fibrosis (*F* 0–2). There were 23 (23%) patients with grade 1 steatosis, 29 (27 %) had grade 2 or 3 steatosis, and 52 (50%) had no steatosis.

The histological findings are summarized in Table 2.

**Table 2:** Histologic fibrosis score of 104 consecutive patients with chronic liver disease who underwent PUD, standard US, TE, and liver biopsy to diagnose severe fibrosis/cirrhosis

	Mean	Median (range)
Biopsy core length (cm)	2.5	2.1 (1.5–4.8)
Portal spaces (n.)	19.5	17.5 (12–40)
Fibrosis score	***n***	**%**
F 0-1	51	49
F 2	24	23
F 3	20	19
F 4	8	8
Not estimable	1	1
Grading score		
A0	14	13
A1 A2	38 31	37 30
A3	21	20
Steatosis grade		
0 1 2–3	52 23 29	50 23 27

The first operator found LSN positivity at PUD examination in 32 patients, among whom 22 had a Metavir score F≥3. The second operator found LSN positivity in 29 patients, among whom 23 had a Metavir score *F* ≥ 3. For the diagnosis of advanced fibrosis and compensated liver cirrhosis at histology, the diagnostic accuracy of PUD, standard ultrasound, and TE are expressed by the diagnostic estimates in Table 3.

**Table 3:** Diagnostic accuracy estimates of severe fibrosis/cirrhosis in 104 consecutive patients with chronic liver disease who underwent pocket-ultrasound device (*PUD*), standard ultrasound (*US*), transient elastography (*TE*), and liver biopsy

Technique	Diagnosis	Sensitivity (95% CI)	Specificity (95% CI)	LR+ (95% CI)	LR- (95% CI)	PPV (95% CI)	NPV (95% CI)	AUROC (95% CI)
PUD	*F* ≥ 3	78.6 (59.0–91.7)	90.7 (81.7–96.2)	8.42 (4.05–17.49)	0.24 (0.12–0.48)	75.9 (56.5–89.7)	91.9 (83.2–97.0)	0.85 (0.76–0.93)
	*F* = 4	87.5 (47.3–99.7)	76.8 (67.1–84.9)	3.78 (2.41–5.93)	0.163 (0.02–1.02)	24.1 (10.3–43.5)	98.6 (92.7–100)	0.82 (0.69–0.95)
Standard US	*F* ≥ 3	89.3 (71.8–97.7)	89.3 (80.1–95.3)	8.37 (4.29–16.32)	0.120 (0.04–0.35)	75.8 (57.7–88.9)	95.7 (88.0–99.1)	0.89 (0.83–0.96)
	*F* = 4	87.5 (47.3–99.7)	72.6 (62.5–81.3)	3.2 (2.10–4.86)	0.172 (0.02–1.08)	21.2 (9.0–38.9)	98.6 (92.3–100)	0.80 (0.67–0.93)
TE	*F* ≥ 3	71.4 (51.3–86.8)	88.0 (78.4–94.4)	5.95 (3.09–11.47)	0.325 (0.18–0.58)	69.0 (49.2–84.7)	89.2 (79.8–95.2)	0.80 (0.70–0.89)
	*F* = 4	87.5 (47.3–99.7)	90.5 (82.8–95.6)	9.24 (4.70–18.13)	0.138 (0.02–0.86)	43.8 (19.8–70.1)	98.9 (93.8–100)	0.89 (0.76–1.00)

The median TE value in patients with F0 was 4.8 kPa, 7.5 kPa in those with *F* ≥ 2, and 11.7 kPa in those with *F* ≥ 3. Statistically significant differences for stiffness values were found between F0–F1 and F2 (*p* = 0.027), between F0–F1 and F3–F4 (*p* < 0.0001), and between F2 and F3–F4 (*p* = 0.0029).

## Discussion

The present study has shown that PUD has excellent reproducibility in assessing LSN even with operators of different experience. Reproducibility is a sine qua non pre-requisite for the adoption of any test. Notably, PUD has performed very well in excluding advanced liver disease and thus can be used as a first-line tool for screening patients to undergo more invasive techniques.

On considering the primary outcome of diagnostic accuracy, PUD has shown high sensitivity as compared to the histological reference standard for cirrhosis (Sn 87.5%) and comparable to TE, even if the latter showed higher specificity at around 90%. Regarding the diagnosis of advanced fibrosis, PUD and TE have shown similar results. The good diagnostic accuracy of PUD in identifying LSN, expressed as good sensibility to rule advanced fibrosis out, was similar to standard US and TE, with histology as the term of comparison. Therefore, considering the lower cost and easy-to-carry convenience of PUD, its use can be recommended at hospital bedside or in a primary care setting, to perform as a screening test to select patients at lower risk. More expensive and invasive techniques, such as CT or MRI, can be reserved to patients at higher or indeterminate risk, as a confirmation test in a secondary or tertiary care setting. The false-negative result at PUD and standard US was a man with morbid obesity (BMI > 35) who presented with severe steatosis (70–80%) at histology. False-positive results, again super-imposable for PUD and standard US, occurred at the evaluation of the liver structure as “granular,” in the absence of advanced fibrosis, and probably attributable to an initial deposition of fibrotic tissue in the more superficial parts of the parenchyma. Such a circumstance might be characteristic of some types of liver disease (vascular, idiopathic portal hypertension), but not that significant from a histological-clinical point of view.

Regarding TE, the estimates of diagnostic accuracy to predict advanced fibrosis and cirrhosis were similar to previous studies on the same cohort [16]. Overall, the sensitivity values were similar to those of the two other US techniques, while TE specificity was slightly higher. It is interesting to note that those patients affected by an important clinical-histological inflammation and by severe cholestasis, which presents falsely higher hepatic stiffness values compared to the actual stage of fibrosis at histology, were correctly assessed through ultrasound techniques, this supporting the notion that the latter methods are not influenced by inflammation nor cholestasis when evaluating fibrosis. Finally, compared to transient elastography, the advantage of ultrasound techniques is that their results are not affected by the possible presence of ascites, usually a sign of cirrhosis and also present in patients with a non-cirrhotic liver. Therefore, ultrasound can be considered better performing in this clinical situation (presence of ascites), compared to TE.

As previously reported in several papers [22–25], the main obstacles to a correct evaluation by ultrasound and elastography methods are related to high BMI. In the present study, the diagnostic accuracy of both PUD and standard US has shown to be reduced in patients with high BMI (≥ 28 kg/m^2^), confirmation of the fact that the main limit for ultrasound remains the increased subcutaneous thickness.

This study has shown some limitations: it applied a 5-degree histology score (METAVIR classification) for staging liver fibrosis regardless of etiology of chronic liver disease, despite this system of classification being used for viral liver diseases. However, in order not to underestimate the degree of fibrosis, in our study the adequacy criteria of the biopsy samples were pre-defined in terms of length and number of portal spaces, and no inadequate sample was collected. Another limit is the size of the sample: the data collected are still limited (only 8 patients who underwent liver biopsy, had cirrhosis at histology), so further studies are needed to confirm these results.

A further limitation we should acknowledge is that the negative predictive value (NPV) strongly depends on the prevalence of the target condition: since we had 8/104 patients with cirrhosis, this low prevalence would ensure good NPV even with a sub-optimal diagnostic test. However, the good performance of the test in a screening strategy is also supported by LR- estimation, providing low values. In fact, the likelihood ratios are independent from pre-test probability, that is the prevalence of the target condition. Nevertheless, the strengths of this study are many: it is a prospective phase II study and a diagnostic accuracy study. It analyzed both diagnostic accuracy and reproducibility. Reproducibility was evaluated between two operators blind to each other’s results. The diagnostic accuracy of the index test (PUD) in identifying LSN (target condition) was compared to histology as the reference standard. There was a single target condition, which was evaluated on the same day with PUD, traditional US, and TE, and with the reference standard of biopsy also performed on the same day. The study population is a population of consecutive patients who were referred to our tertiary referral center for liver biopsy. The population was heterogeneous of different CLD etiologies, on one side this reducing the internal validity of the present study, but on the other side enabling to increase the external validity in a hepatology primary care or general medicine setting.

In conclusion, the key deliverable of this study is the evidence that, even after a short period of training, an operator can rely on PUD to rule out the clinical suspicion of advanced fibrosis/compensated cirrhosis. Thanks to its small size, availability, and low cost, PUD can be used as a screening tool for selecting patients who need to undergo more invasive examination in order to have the presence of advanced liver disease confirmed. As a triage test to immediately exclude advanced stage diseases, PUD can contribute to a reduced prescription of additional tests, especially in liver outpatient primary or secondary care settings, where traditional ultrasound equipment is not always available.

## Supplementary Information

ESM 1 (PDF 296 kb)

## Abbreviations

CDI: Color Doppler imaging
CLD: Chronic liver disease
LSN: Liver surface nodularity
PUDs: Pocket-sized ultrasound devices
TE: Transient elastography
US: Ultrasound

## Declarations

### Guarantor

The scientific guarantor of this publication is Dr. Mirella Fraquelli (Gastroenterology and Endoscopy Unit, Fondazione IRCCS Ca’ Granda Ospedale Maggiore Policlinico, Milano, Italy).

### Statistics and biometry

One of the authors (GC) has significant statistical expertise.

### Methodology

• prospective

• cross-sectional study

• diagnostic

• performed at one institution
